# Algorithms to Model Single Gene, Single Chromosome, and Whole Genome Copy Number Changes Jointly in Tumor Phylogenetics

**DOI:** 10.1371/journal.pcbi.1003740

**Published:** 2014-07-31

**Authors:** Salim Akhter Chowdhury, Stanley E. Shackney, Kerstin Heselmeyer-Haddad, Thomas Ried, Alejandro A. Schäffer, Russell Schwartz

**Affiliations:** 1 Joint Carnegie Mellon/University of Pittsburgh Ph.D. Program in Computational Biology, Carnegie Mellon University, Pittsburgh, Pennsylvania, United States of America; 2 Lane Center for Computational Biology, Carnegie Mellon University, Pittsburgh, Pennsylvania, United States of America; 3 Intelligent Oncotherapeutics, Pittsburgh, Pennsylvania, United States of America; 4 Genetics Branch, Center for Cancer Research, NCI, NIH, Bethesda, Maryland, United States of America; 5 Computational Biology Branch, NCBI, NIH, Bethesda, Maryland, United States of America; 6 Department of Biological Sciences, Carnegie Mellon University, Pittsburgh, Pennsylvania, United States of America; University of California San Diego, United States of America

## Abstract

We present methods to construct phylogenetic models of tumor progression at the cellular level that include copy number changes at the scale of single genes, entire chromosomes, and the whole genome. The methods are designed for data collected by fluorescence *in situ* hybridization (FISH), an experimental technique especially well suited to characterizing intratumor heterogeneity using counts of probes to genetic regions frequently gained or lost in tumor development. Here, we develop new provably optimal methods for computing an edit distance between the copy number states of two cells given evolution by copy number changes of single probes, all probes on a chromosome, or all probes in the genome. We then apply this theory to develop a practical heuristic algorithm, implemented in publicly available software, for inferring tumor phylogenies on data from potentially hundreds of single cells by this evolutionary model. We demonstrate and validate the methods on simulated data and published FISH data from cervical cancers and breast cancers. Our computational experiments show that the new model and algorithm lead to more parsimonious trees than prior methods for single-tumor phylogenetics and to improved performance on various classification tasks, such as distinguishing primary tumors from metastases obtained from the same patient population.

## Author Summary

Cancer is an evolutionary system whose growth and development is attributed to aberrations in well-known genes and to cancer-type specific genomic imbalances. Here, we present methods for reconstructing the evolution of individual tumors based on cell-to-cell variations between copy numbers of targeted regions of the genome. The methods are designed to work with fluorescence *in situ* hybridization (FISH), a technique that allows one to profile copy number changes in potentially thousands of single cells per study. Our work advances the prior art by developing theory and practical algorithms for building evolutionary trees of single tumors that can model gain or loss of genetic regions at the scale of single genes, whole chromosomes, or the entire genome, all common events in tumor evolution. We apply these methods on simulated and real tumor data to demonstrate substantial improvements in tree-building accuracy and in our ability to accurately classify tumors from their inferred evolutionary models. The newly developed algorithms have been released through our publicly available software, FISHtrees.

This is a *PLOS Computational Biology* Methods article.

## Introduction

In this paper, we develop new methods to advance the theory of phylogenetic inference for reconstructing evolutionary histories of cell populations in solid tumors. The work is specifically designed for use in tracking tumor evolution by gain and loss of genomic regions as assessed by multicolor fluorescence *in situ* hybridization (FISH), which measures the copy numbers of targeted genes and chromosomes in potentially hundreds of individual cells of a tumor. This technology was the basis of the earliest methods for phylogenetic reconstruction of single tumors [1], [2]. FISH remains uniquely valuable for such studies because the large number of cells that FISH can profile makes it possible to collect data on enough tumors in enough detail to build cell-by-cell phylogenies for populations of tumors and begin to study the common features of these phylogenies. In the present work, we specifically extend our previously developed inference algorithms to encompass a more complicated but realistic model of evolution of FISH probe counts, accounting for gain and loss of genetic material at the level of single gene probes, multiple probes on a single chromosome, or a probe set distributed across the whole genome. We demonstrate the value of these algorithmic improvements to more accurate phylogenetic inference and improved effectiveness of the resulting phylogenies in downstream prediction tasks.

The present work adds to the growing list of phylogenetic methods in cancer modeling, which were reviewed through 2008 in [3]. These include methods for analyzing comparative genomic hybridization (CGH) or other genetic gain/loss data in a single tumor type [4]–[11], for defining the cell type lineage of single tumors [1], [2], [12], , for organizing a taxonomy of tumor types [14], for reconstructing a partial order of genetic changes in multiple samples from one patient [15], and for reconstructing progression from cell types inferred from bulk genomic assays [16]. Recent high-throughput sequencing studies have also used ad hoc phylogenetic methods to infer putative tumor progression scenarios, e.g., [17]–[20]. Like many of these methods, the present work is aimed at building tree models that provide a proposed partial order on the observed cell states, a strategy motivated originally by the work of Fearon and Vogelstein, proposing a linear order for four types of events in colorectal cancer and associating each event with a tumor stage [21]. Other ordering methods have been proposed, mostly for CGH or breakpoint data [15], [22]–[28] and, more recently, sequencing data [29], [30].

The present work specifically advances the reconstruction of phylogenetic histories of single tumors from intratumor cellular heterogeneity data. The use of phylogenetic methods to reconstruct histories of single tumors was first developed in our prior work [1], [2] by taking advantage of the ability of FISH to profile genetic changes in large numbers of single cells, allowing one to survey hundreds of cells per tumor in populations of tens of tumors [31]. This early work showed that even small numbers of markers could reveal numerous genetically distinct cell populations in single tumors, which could be resolved by phylogenetic inference to reveal multiple distinct pathways of progression between tumors and even within single tumors. Numerous studies since then, using multicolor FISH [2], [31]–[36] and, more recently, single-cell sequencing [19], [37]–[39] have greatly increased our ability to identify distinct cell populations and, in the process, revealed far more extensive intratumor heterogeneity than had been suspected prior to 2010 (reviewed in [40]). The repeated observation of intratumor heterogeneity has necessitated a reconsideration of Nowell's [41] theory that tumors evolve clonally, showing that a tumor may contain many subpopulations relevant to the clinical prognosis of the patient [42] and that rare subpopulations may be more relevant to prognosis than the most common ones [43]. Furthermore, a simulation study has suggested that methods based on average copy number data perform poorly when there is substantial intratumor heterogeneity [44]. Such findings suggest a need for improved methods for organizing the dozens or hundreds of observed cell states in single tumors to infer the evolutionary processes that produced them.

Despite extensive work on tumor phylogenetics, however, the study of algorithms for reconstructing tumor evolution from large numbers of single cells has lagged far behind advances in data generation. The standard in practice for single-cell tumor phylogenetics remains the use of simple generic phylogeny algorithms (e.g., neighbor-joining [45]) that are not designed to model the patterns of copy number changes one would expect from evolution by chromosome abnormalities that largely drive tumor evolution. Until recently, algorithms designed specifically for inferring phylogenies of single tumors from FISH data have been limited to just a few probes per cell and lacked robust, publicly available software implementations [1], [2], [34]. In prior work [46], we developed algorithms to find copy-number phylogenies for in principle arbitrary numbers of probes and cells. That work, however, was itself limited to a simple model in which tumor cells evolve by events of gain or loss of a single copy number of a single probe at each mutation step. In real tumors, gene copy numbers can change due to a variety of mechanisms, including:

1. Single gene duplication/loss events (SD), in which one copy of a genetic region covered by a single probe is gained or lost.
2. Chromosome duplication/loss events (CD), in which entire chromosomes are unequally distributed among daughter cells during mitosis along with potentially several probes.
3. Whole genome duplication events (GD), in which a cell fails to divide during mitosis leading to doubling of all genetic material and all probe counts.

**Figure 1:**
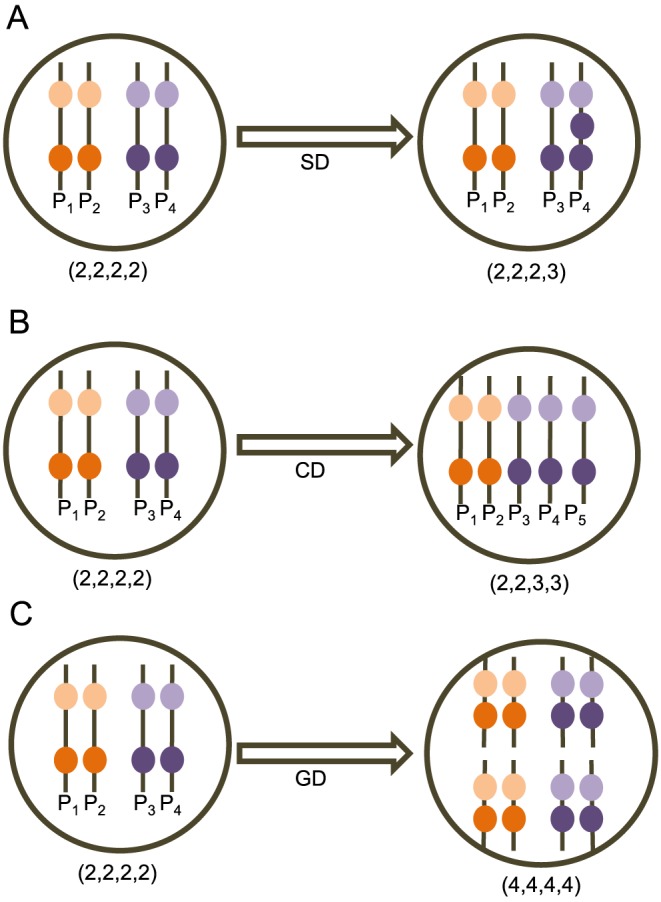
Example showing the three mechanisms of copy number changes in a hypothetical cell. A copy number profile of four genes is shown as an ordered set for homologous chromosome pairs 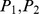 and 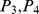 respectively, where the gene located on the top position in the chromosome precedes the gene located on the bottom position in the ordering. After the (A) Single gene duplication event, the copy number of a gene located on 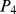 gets increased by 1. After the (B) Single chromosome duplication event, the chromosome 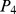 gets duplicated and the cell has one extra copy of that chromosome as chromosome 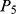. After the (C) Whole genome duplication event, all the chromosomes are duplicated and the total number of chromosomes in the daughter cell is twice the number of chromosomes in the mother cell.

These events are illustrated schematically in Figure 1. While more complex probabilistic models of tumor evolution have been developed for inference of small phylogenies, with approximately ten taxa per tumor corresponding to distinct biopsies (e.g., [47]), the class of inference algorithms such models require would not be expected to scale to phylogenies of hundreds of single cells per tumor such as those examined in the present work.

The work presented here seeks to fill this need for scalable phylogenetic algorithms capable of fitting more realistic models of tumor-like evolution to data sets of hundreds of single cells per tumor. We improve on our prior work for inferring tumor evolutionary models considering only SD events [46] to now include CD and GD events, which are also frequently observed in tumor progression. We specifically focus on the problem of accurately inferring evolutionary distances between distinct cells in terms of maximum parsimony combinations of SD, CD, and GD events. The major contributions of the work are:

1. algorithms to compute minimum evolutionary distances 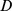 between pairs of cell states in terms of SD and CD events and in terms of SD, CD, and GD events;
2. a heuristic Steiner tree method based on the median-joining method [48] and our prior work on SD-only inference [46];
3. software implementation of the new methods to compute 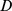 and use of those methods to construct tumor progression trees;
4. evaluation of the new methods on simulated data, which shows that they do better than the SD-only approach at recovering simulated tree topologies;
5. application of the methods to published data on cervical cancer (CC, [49]) and breast cancer (BC, [36]);
6. demonstration of improved ability to classify tumor types from phylogenetic features using a strategy in the spirit of the genomic progression scores (GPS) of Rahnenführer et al. [50].

The new methods are implemented in version 2 of our software FISHtrees (ftp://ftp.ncbi.nlm.nih.gov/pub/FISHtrees). The work addresses a critical need in modern cancer research for algorithms capable of inferring evolutionary trajectories of hundreds of single cells per tumor under plausible models of evolution including both gene-specific and chromosome abnormalities that are central drivers of true tumor evolution.

## Results

**Figure 2:**
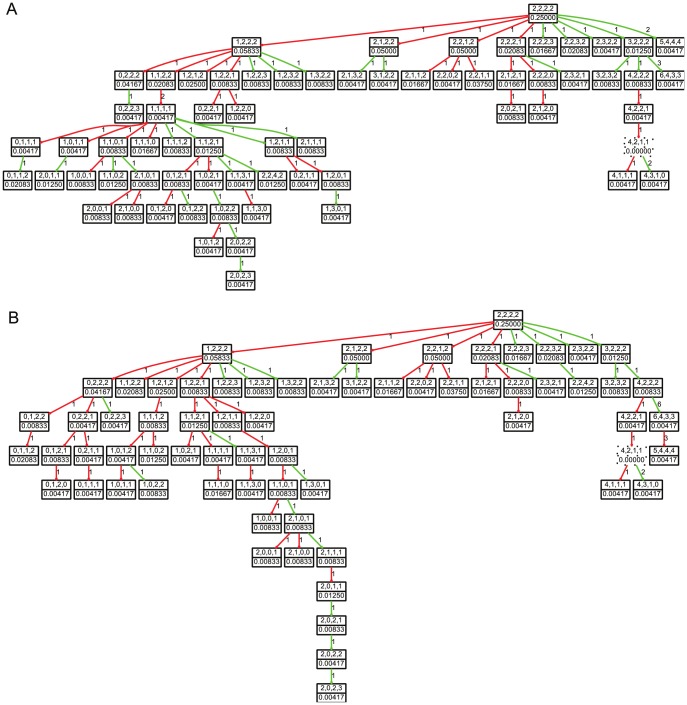
Phylogenetic trees showing tumor progression in a cervical cancer patient. Trees are built considering (A) all of SD, CD and GD and (B) only SD model of tumor evolution. Each node represents a configuration of the four gene probes *LAMP3*, *PROX1*, *PRKAA1* and *CCND1*. Nodes with solid and dotted borders represent cells present in the collected sample and inferred Steiner nodes respectively. Green and red edges model gene gain and gene loss, respectively. The weight value on each edge connecting two nodes 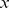 and 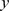 is the distance between the states of 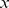 and 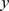, computed using the particular model of tumor progression under consideration. The weight on each node describes the fraction of cells in the sample with the particular copy number profile modeled by that node; Steiner nodes are assigned weight 0.

We used data collected from cervical cancer (CC) [49] and breast cancer (BC) [36] patients to evaluate our methods. Figure 2(A) shows a tumor progression tree inferred from one of the cervical cancer samples. For comparison, Figure 2(B) shows a progression tree inferred on the same sample using our prior SD model [46]. Visual inspection shows that large regions of the two trees are identical but that allowing CD and GD events leads to some rearrangement and a reduction in tree depth and overall size. Next we evaluate the changes induced by adding SD, CD and GD events, using simulated data to show effectiveness of the methods in finding more parsimonious solutions to the broader model and using the real CC and BC data to show the biological relevance of the improvements. We further show that our algorithms infer trees with higher accuracy than the prevailing alternative algorithms for single-tumor phylogenetic inference. Finally, we perform statistical experiments to evaluate the effects of tumor sample size on the performance of our tree building algorithm.

### Simulation experiments

To measure accuracy of the methods for FISH datasets with a known ground truth, we generated a dataset of 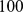 trees with six probes, two of which were treated as being on the same chromosome. Each tree was generated by starting from a diploid root node and executing a branching process in which each node was recursively assigned a number of children drawn from a geometrically distributed random variable with mean 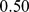. Each child was distinguished from its parent by selecting an SD, CD, or GD event with probability 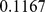 for each of the six possible SD events, 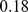 of a CD event, and 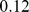 of a GD event. This process terminated when all leaf nodes had been assigned zero children by the sampling. We then generated simulated FISH data for each tree by uniformly sampling 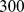 cells from the nodes in this topology. The simulated data corresponds to counts of probes for each sampled cell in the tree. We applied Algorithm 3 (see Methods) to find a minimum-cost tree for each of four event models: (i) SD only, (ii) SD and CD, (iii) SD and GD, and (iv) SD, CD and GD.

We quantified the accuracy of tree inference by comparing each simulated true tree to its corresponding inferred tree derived from the sampled cells. This assessment was performed at the level of accuracy of tree edges by the following procedure:

1. We pruned the real tree so as to remove any subtree for which no cell in the tree was sampled. This step was intended to avoid penalizing for “impossible” inferences of subtrees unsupported by any data.
2. We computed a maximum matching of edges between the real subtree and the inferred tree, with each pair of edges weighted by the maximum number of nodes in agreement between the corresponding parts of the bipartitions that the two edges define [46], [51]. We used the Hungarian algorithm [52] for computing the maximum matching (applying the function“Hungarian” by Alexander Melin from the Matlab Central File Exchange).
3. We calculated a reconstruction error 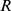 of the inferred tree using the following formula:

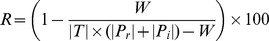
where 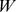 is the weight of the maximum matching, 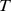 is set of taxa in common between the real and inferred trees, and 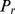 and 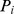 represent the sets of nontrivial bipartitions in the real and inferred trees, respectively.

Intuitively, this formula measures the fractional agreement between bipartitions of the trees relative to the total number of bipartitions. We use a matching-based formula, rather than the more familiar Robinson-Foulds metric [53], both because of its greater sensitivity to small changes in trees and because the Robinson-Foulds measure is not defined for trees with different node sets. We also note that we use a different normalization factor than in our prior work [46], normalizing essentially by the total number of edges between the two trees, to control properly for the fact that different inference methods may infer different numbers of tree edges. The reconstruction error 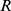 ranges in value from 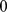, if the real and inferred trees are isomorphic, to an upper bound of 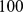 in the limit of complete disagreement.

**Figure 3:**
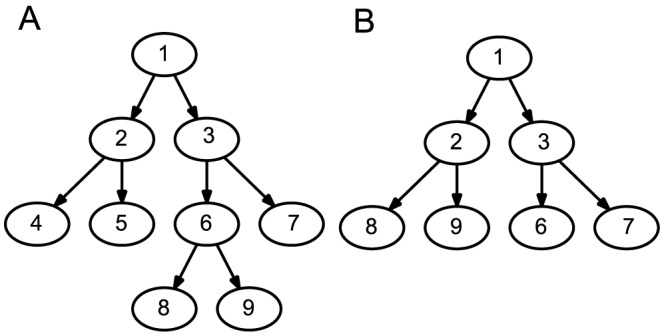
Example simulated and inferred trees illustrating key terms in the formula for calculating the reconstruction error. (A) A hypothetical simulated ground truth tree on the set of taxa 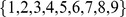. (B) Example inferred tree built on the sampled set of taxa 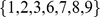 on the dataset resulting from the ground truth tree.



To illustrate the meanings of the terms of the equation for 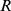, we present a simple example using a hypothetical ground truth and an inferred tree presented in Figure 3(A) and Figure 3(B), respectively. The set of nontrivial bipartitions in the ground truth are

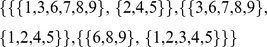
and the nontrivial bipartitions in the inferred tree are



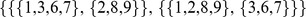



If we apply the matching algorithm on these two sets of bipartitions, the first and second bipartitions in the ground truth tree are matched with the first and second bipartitions in the inferred tree, respectively. The weight 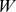 of the matching is 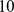. The number of common taxa between these two datasets is 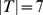. The total number of nontrivial bipartitions in the real and inferred trees are 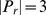 and 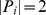. Plugging these values into the equation for 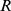, we calculate 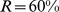.

**Figure 4:**
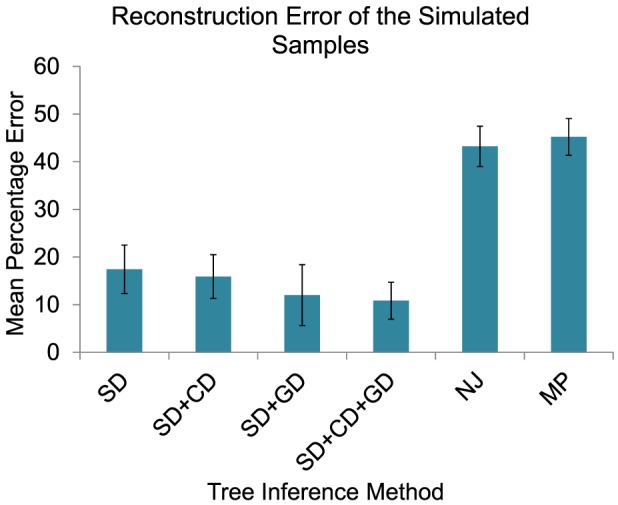
Accuracy of phylogenetic inference on simulated copy number data for varying algorithms. Variants of our phylogenetic algorithms and two competing methods from the literature were applied to simulated FISH datasets describing evolution by combinations of single-gene (SD), chromosome (CD), and whole-genome (GD) duplication and loss events. Results are reported for inference by our methods from 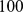 simulated trees, allowing for SD events alone, SD+CD events, SD+GD events, and SD+CD+GD events. We compared these results to inference by neighbor-joining (NJ) and pure maximum parsimony (MP) as implemented in MEGA, version 6. Accuracy is assessed by mean reconstruction error of bipartitions between true and inferred trees. Error bars show plus or minus one standard deviation across the samples for each method.

A comparison of the four models is presented in Figure 4. The SD model showed 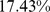 reconstruction error with standard deviation (s.d.) of 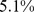 across the 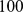 trees. The SD+CD model yielded 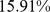 error with s.d. 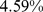. SD+GD yielded 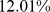 error with s.d. 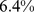. The full SD+CD+GD model yielded 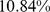 error with s.d. 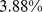. Collectively, the results suggest that one can reconstruct reasonably accurate trees even from the SD-only model, despite the fact that the trees were generated from a model of all three event types, although accuracy improves with each event type added. Accounting for GD events made a larger difference in accuracy than accounting for CD events, presumably because a missed GD event might require many SD or CD events to explain it, while a missed CD event could be explained with just two SD events. The reconstruction error for the full model is reduced by more than 1.7-fold relative to the SD-only model considered in our prior work.

We further compared these results to those derived using generic phylogenetic methods that have been used in much of the single tumor phylogenetics work to date [16], [54]. We tested the accuracy of reconstruction of the 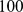 simulated trees described above using generic neighbor joining (NJ) with Euclidean distance and pure maximum parsimony (MP) treating copy numbers as arbitrary characters, approaches chosen because they have been the primary alternatives to our specialized algorithms in the single-tumor phylogeny literature. We omit here comparison to more complicated Bayesian phylogenetic models (e.g., [47]) because such approaches are not scalable to the numbers of cells we examine. We then used the weighted matching based similarity method, described above, to calculate the mean percentage reconstruction error 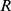 between the inferred and the ground truth trees. The mean reconstruction errors for NJ and MP were 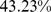 (s.d. 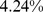) and 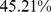 (s.d. 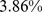), respectively, in contrast to the error of 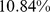 (s.d. 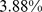) for the SD+CD+GD algorithm proposed here. The test thus demonstrates that when the underlying evolutionary process includes cancer-like chromosome abnormalities, errors are substantially reduced by using an algorithm designed for that model relative to standard off-the-shelf algorithms still widely used for single-tumor phylogenetics work.

We performed additional experiments to evaluate the effects of different evolutionary parameters on the accuracy of inference of tumor progression trees by FISHtrees. For this experiment, we selected five different combinations of probabilities of SD, CD and GD events for generating the ground truth trees and then used SD, SD+CD, SD+GD and SD+CD+GD models to infer the tumor phylogenies. These data sets again each used six probes with two of the six on a common chromosome. The selected five combinations of (SD,CD,GD) event probabilities are: 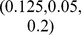, 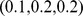, 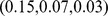, 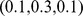 and 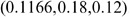. These combinations of event probabilities were chosen to yield trees of comparable complexity to the real data while producing test sets enriched in distinct combinations of the three event types. They thus allow us to consider how robust our algorithms are to contributions from each of the three event types, singly or in combination. We report the reconstruction error for 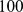 trees for each of these combinations of event probabilities in Table 1. These results again show that accuracy improves with each event type added. When the probability of SD events is high (as in combination 3), the SD model results in highly accurate trees (mean reconstruction error of 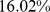 with s.d. 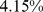). Accounting for GD events in combination with SD events always result in larger improvement in the reconstruction error in comparison to the SD+CD models, even when the CD events are very frequent (as in combinations 2 and 4). Finally, accounting for GD events in combination with SD and CD events results in the largest improvements when the probability ratio of GD events to SD+CD events is highest, as can be seen from comparison of parameter sets 1 and 2.

**Table 1:** Comparison of mean percentage reconstruction error (with standard deviation) of different phylogeny models on simulated data for different combinations of SD, CD and GD event probabilities.

Probabilities of (SD,CD,GD) Events	SD	SD+CD	SD+GD	SD+CD+GD
(0.125,0.05,0.2)	17.97(4.49)	16.89(4.32)	9.85(3.51)	9.25(4.18)
(0.1,0.2,0.2)	25.58(4.50)	21.82(3.98)	13.81(3.62)	10.96(3.99)
(0.15,0.07,0.03)	16.02(4.15)	14.96(4.16)	11.92(4.29)	11.71(4.77)
(0.1,0.3,0.1)	23.13(4.37)	20.02(4.50)	15.43(4.60)	13.42(4.64)
(0.1166,0.18,0.12)	17.43(5.10)	15.91(4.59)	12.01(6.40)	10.84(3.88)

Mean percentage reconstruction error on 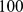 simulated samples are shown for four tree-building models considering (i) SD, (ii) SD+CD, (iii) SD+GD and (iv) SD+CD+GD across five different combinations of SD, CD, and GD probabilities.

Next, we performed simulation tests to evaluate the effects of non-uniform distributions of cells across different levels of the trees on the performance of our tree inference method. In our initial simulation experiments described above, we assumed that observed cells were sampled uniformly across clones. In real tumors, the distribution of cells would not typically be uniform due to differences in age and fitness of clones. In order to test robustness of our method to non-uniformity of clone frequencies, we sampled the cells following a non-uniform model in which the sampling frequency of a clone varies geometrically with its depth in the tree with a parameter 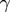. We used values of 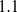 and 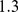 for 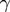 in our experiments. When 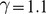, 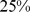 of the total cells are located in the first three levels of the trees, while for 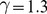, this fraction is 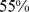. We generated 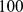 trees in each case with probabilities of SD, CD and GD events fixed at 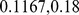 and 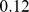. We again used SD, SD+CD, SD+GD and SD+CD+GD models to infer the tumor progression trees. We present the results from this experiment in Table 2, where we also show the results from the uniform sampling of the cells. Additionally, we report the results on the trees inferred using NJ and MP for these three different cell distributions. From the table, we can see that the reconstruction error increases with increasing 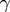 for all methods. The SD+CD+GD model, however, shows the best performance among all the models for all three values of 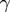 and the least loss of performance with increasing 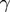.

**Table 2:** Comparison of mean percentage reconstruction error (with standard deviation) of different phylogeny models on simulated data for different sampling distributions of the cells.

Distribution	SD	SD+CD	SD+GD	SD+CD+GD	NJ	MP
Uniform	17.43(5.10)	15.91(4.59)	12.01(6.40)	10.84(3.88)	43.23(4.24)	45.21(3.86)
Skewed ( 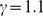 )	22.74(4.49)	19.09(4.47)	14.75(4.64)	11.92(4.64)	47.00(3.76)	47.38(3.72)
Skewed ( 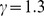 )	29.93(7.37)	26.35(6.56)	18.89(7.24)	15.36(6.78)	50.63(5.89)	50.32(5.74)

Mean percentage reconstruction error on 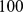 simulated samples are shown for six tree-building models considering (i) SD, (ii) SD+CD, (iii) SD+GD, (iv) SD+CD+GD (v) NJ and (vi) MP when the sampling distribution of cells is varied.

Finally, we performed simulation experiments to understand the effects of varying the numbers of chromosomes with multiple probes. We created a simulated dataset of 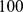 trees with eight probes where two pairs of probes each reside on two different chromosomes and the remaining four probes reside on four separate chromosomes. The probabilities of each of the SD, CD and GD events were fixed at 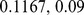, and 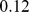, respectively. We report the results from this experiment in Table 3, which compares the results from this experiment with our earlier result using only a single chromosome with two probes and four other probes located on separate chromosomes. The table shows that inclusion of the extra possible CD event results in higher accuracy for all the models except for the SD only model. The performance drop in the SD model is expected, as it would require more SD events to explain a greater number of missed CD events. The highest gain in performance is observed for SD+CD+GD model. These results show that our algorithm will tend to yield comparatively more advantage over the earlier work with more complicated scenarios of sharing probes across chromosomes, suggesting its utility will increase as improvements in technology allow for larger probe sets.

**Table 3:** Comparison of mean percentage reconstruction error (with standard deviation) of different phylogeny models on simulated data for two different probe settings.

Number of Chromosomes with 2 Genes	SD	SD+CD	SD+GD	SD+CD+GD
1	17.43(5.10)	15.91(4.59)	12.01(6.40)	10.84(3.88)
2	19.01(5.61)	15.65(5.26)	11.49(4.18)	8.94(3.46)

Mean percentage reconstruction error on 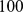 simulated samples are shown for four tree-building models considering (i) SD, (ii) SD+CD, (iii) SD+GD and (iv) SD+CD+GD for two different cases when the number of chromosomes harboring two genes is 1 or 2.

### Application to real cervical and breast cancer data

We applied the algorithm to two sets of real data:

- A set of CC [49] FISH data consisting of 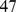 samples organized into 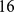 primary samples of metastatic patients, 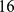 paired metastasis samples from the same patients, and 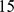 primary samples from patients who did not progress to metastasis. Each sample consisted of 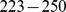 cells profiled on four FISH probes: *LAMP3* (Entrez Gene Id 27074) [55], *PROX1* (5629) [56], *PRKAA1* (5562) [57] and *CCND1* (595) [58]. All of these four genes are oncogenes, which typically show copy number gains in tumor cells. Each of the genes belongs to a distinct chromosome.
- A set of BC [36] FISH data consisting of 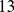 paired (from the same patient) ductal carcinoma in situ (DCIS) and invasive ductal breast carcinoma (IDC) samples with 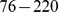 cells per sample profiled on eight FISH probes: *COX-2* (5743) [59], *MYC* (4609) [60], *CCND1* [58], *HER-2* (2064) [61], *ZNF217* (7764) [62], *DBC2* (23221) [63], *CDH1* (999) [64] and *TP53* (7157) [65]. The first five genes in this list are oncogenes and the last three genes are tumor suppressors. In tumor cells, tumor suppressors are typically associated with loss in copy numbers.

Among the eight genes in the BC dataset, *DBC2* and *MYC* reside on chromosome 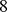 and *HER-2* and *TP53* reside on chromosome 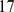. The other four genes belong to distinct chromosomes. The oncogene Cyclin D1 (*CCND1*), which plays a role in many solid tumor types, is in both the BC and CC datasets. However, in some other tumor types, such as oral cancer, *CCND1* is part of a larger region with recurrent copy number gains on chromosome 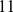 and other nearby genes have also been suggested to play a role in oncogenesis [66].

**Figure 5:**
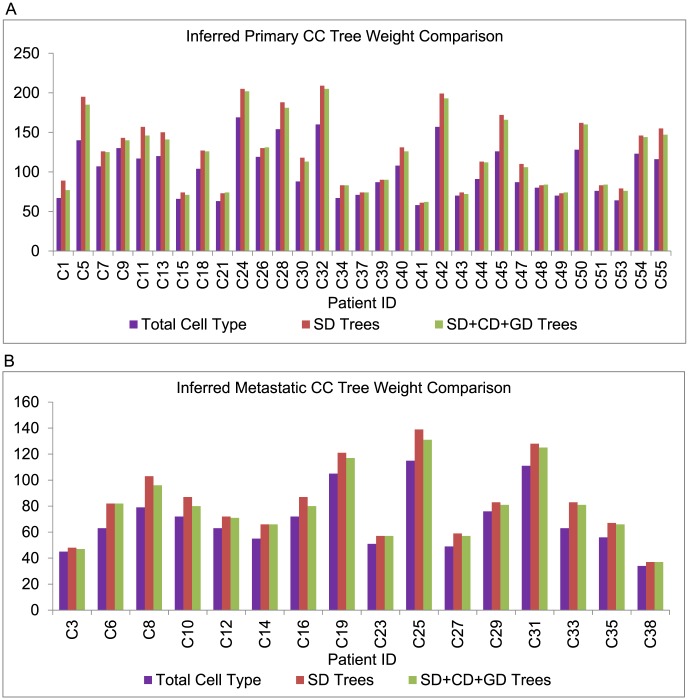
Parsimony score comparison on the CC samples. Comparison of (A) Primary and (B) Metastatic CC tumor progression tree weights built considering only SD and combined SD, CD and GD models. “Total Cell Type” refers to the total number of unique probe copy number configurations in the dataset, providing a lower bound on the minimum possible parsimony score for a given data set.

**Figure 6:**
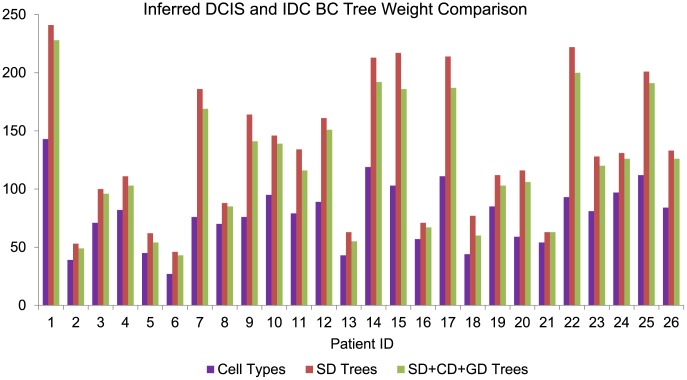
Parsimony score comparison on the BC samples. Comparison of DCIS (id 1–13) and IDC (id 14–26) BC tumor progression tree weights built considering only SD and combined SD, CD and GD models. “Cell Types” refers to the total number of unique probe copy number configurations in the dataset, providing a lower bound on the minimum possible parsimony score for a given data set.

We evaluated the SD+CD+GD method by its effectiveness in reducing the parsimony score (total number of mutation events) of the resulting trees relative to the prior SD-only model. With the primary CC samples, the SD+CD+GD method found a lower-cost tree in 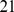 of 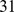 cases, a tree of equal weight in 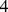 cases, and a higher-cost tree in 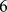 cases. In each case of increased weight, the increase was by 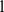 and appears to result from the subtree regrafting heuristic used in handling GD events (see Methods). These results suggest that the heuristic tree search may more often yield a suboptimal result for the SD+CD+GD model than it does for the SD-only model. The benefit of the more realistic model, however, outweighs the cost of this suboptimality in a large majority of instances. For trees derived from metastatic samples, 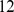 of 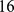 trees had lower weight for the full SD+CD+GD model and the remainder all had equal weight for the two models. Metastatic data sets tend to have fewer distinct cell types than do primary trees and thus may represent an easier optimization challenge. For the BC samples, 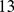 of 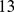 DCIS (samples 1–13) and 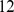 of 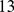 IDC (samples 14–26) had lower weight for the full model, with the remaining one sample having equal weight. Parsimony scores by tree are provided in Figures 5 and 6.

**Figure 7:**
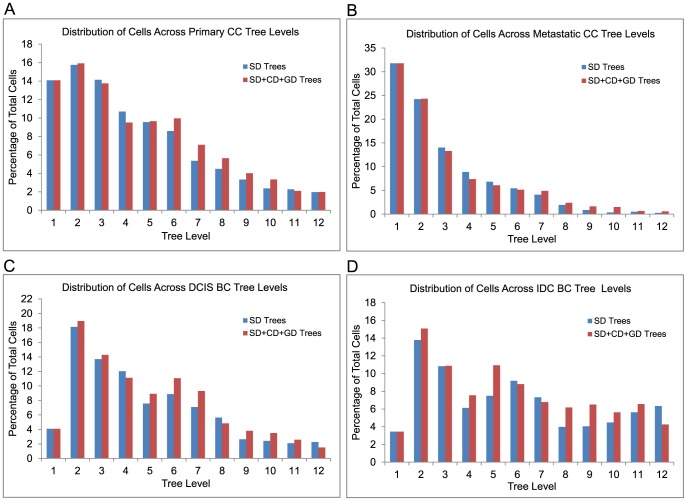
Distribution of cells across different levels of tumor phylogenies. Distribution of cells across different levels are shown for (A) Primary and (B) Metastatic CC, and (C) DCIS and (D) IDC BC tumor progression trees.

We next evaluated effects of the improved model on overall tree topology, based on results of our prior work [46] that tree topology can significantly distinguish trees drawn from distinct progression stages of a given tumor type, with possible implications for the varying balance of diversification and selection acting on different stages of tumor progression. Figure 7 quantifies the topology for each sample set based on fractions of cells inferred at each tree depth from 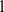 to 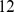. The figure shows similar qualitative trends for both SD and SD+CD+GD methods, although with small quantitative differences. For example, both SD and SD+CD+GD trees recapitulate a tendency for CC primary trees to show relatively broad topology (Figure 7(A)) while CC metastatic trees prune rapidly beyond the first few tree levels (Figure 7(B)). There is, however, an overall shift to lower depth in the SD+CD+GD trees. For CC primary trees, 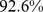 of cells are located in the first 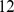 tree levels for SD versus 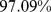 for SD+CD+GD. For CC metastatic, 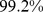 of cells are located in the first 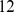 tree levels for SD versus 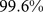 for SD+CD+GD. For BC, the comparable numbers of cells in depths 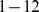 are 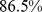 for SD versus 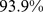 for SD+CD+GD in DCIS and 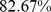 for SD versus 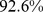 for SD+CD+GD. These results suggest that the overall tree topology is not greatly sensitive to the combination of event types, although there is a noticeable shift towards lower depth in the full model.

An additional evaluation was possible for the BC trees, because for the BC data, a probabilistic model and expert annotation based on two additional centromere probes made it possible to estimate the cell ploidy [36], which we define as the mode among the number of copies of the twenty-two autosomal chromosomes in a cell. Each cell in that dataset is thus annotated with an expert-curated overall ploidy estimate. We used these ploidy estimates to validate our inference of GD events based on whether edges assigned to GD events in our trees correspond to doubling of annotated ploidy. The percentage agreement by edge between GD events and annotated doubling in ploidy is 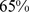 across DCIS trees and 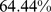 across IDC trees. In 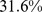 of all inferred GD events, at least one endpoint of the corresponding edge is a Steiner node, and the uncertainty among whether a GD event occurred prior to or after the emergence of the Steiner node may explain why the per-edge agreement is not higher. Nonetheless, the data support the conclusion that inferred GD events are correct in a majority of cases.

As a final step, we repeated an approach developed in our prior work [46] to both validate the biological relevance of the trees and develop a practical application of them by treating the trees as sources of features for classification tasks applied to the CC data. For this purpose, we developed several sets of quantitative features based on inferred trees as well as comparative features derived from raw FISH probe counts. We used the following set of tree-based features:

1. Edge count: 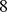 features corresponding to fraction of progression tree edges showing gains and losses of each gene.
2. Tree level cell percentage: 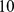 features corresponding to the fraction of cells at each of the first 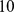 levels for the progression trees.

We omitted a third feature set, bin count, used in our prior work because it is not easily comparable between SD and SD+CD+GD trees. We compared these features to four features derived directly from FISH probe counts without reference to the trees:

1. Mean gain and loss of individual genes.
2. Maximum copy number of individual genes.
3. An information theoretic measure, Shannon index [67]. For each gene, each combination of gene copy number and cellular ploidy represents a species. If we denote the frequency of species 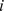 among all tumors by 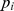, then Shannon index is given by the formula 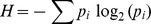.
4. Simpson's index [67], which is defined as 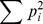.

We used each feature set as input to the Matlab support vector machine (SVM) classifier with a quadratic kernel using 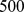 rounds of bootstrap replicates per test with leave-one-out cross-validation to compute mean and standard deviation of accuracy. We used Matlab functions “svmtrain” and “svmclassify” for training and testing of the SVM classifier.

We then applied these methods for three classification tasks: (i) distinguishing primary samples that progressed to metastasis from their paired metastatic samples, (ii) distinguishing all primary samples from all metastatic samples, and (iii) distinguishing primary samples that metastasized from primary samples that did not metastasize. The first two tasks are relevant to identifying features that help us understand the differences in evolutionary mechanisms of primary and metastatic samples. The third is intended to model an important practical problem in cancer treatment: determining whether a given primary tumor will metastasize.

**Figure 8:**
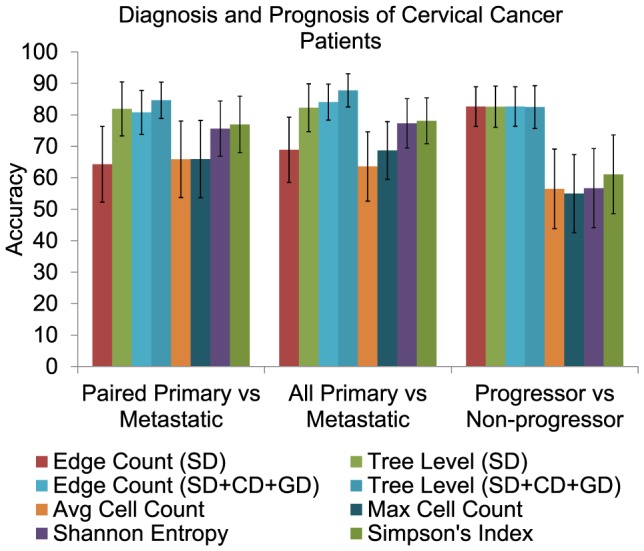
Classification results on the CC dataset. Prediction accuracy on three different classification tasks of CC samples of an SVM classifier using tree-based and cell-based features. Each of the two tree-based features, edge count and tree level cell percentage, is derived from phylogenetic trees built using two different models of tumor progression, namely SD and combination of SD, CD and GD. Two cell-based features, average gain/loss and maximum copy number of each gene, and two information theoretic measures of cell heterogeneity, Shannon entropy and Simpson's index, are used.

Figure 8 shows results on each task. For task (i), allowing SD+CD+GD events increased accuracy relative to SD trees from 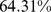 to 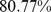 for edge counts and from 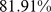 to 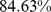 for tree level cell count. The SD+CD+GD tree level cell count was the most effective of all features, tree-based or not. For task (ii), we similarly saw a substantial improvement in prediction accuracy for SD+CD+GD trees relative to SD trees. Classification accuracy improved from 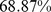 to 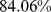 for edge count features and from 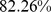 to 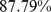 for tree level features. In this case, both SD+CD+GD tree feature sets outperformed all other features sets, tree-based or otherwise. These results provide an indirect validation that using a more general tree model gets closer to the biological ground truth. For task (iii), we saw no improvement, with identical results for SD and SD+CD+GD trees for either feature set. All tree-based feature sets significantly outperformed all non-tree-based feature sets for this task. We conclude that the more realistic evolutionary models appear not to reveal any more information to the classifiers for predicting which primary samples will go on to metastasize than the SD trees, which were already quite effective for that task.

### Dependence on data size

A key advantage of FISH for profiling tumor heterogeneity is that it makes it cost-effective to profile much larger numbers of cells than alternatives such as single-cell sequencing. To assess the practical importance of this advantage, we asked two related questions: (1) how many cells do we need per tumor to accurately reconstruct single-cell phylogenies and (2) how many tumors do we need to examine to identify reproducible, statistically significant features across trees.

We first assessed the number of cells needed per tumor by using our first simulated dataset of 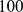 trees described above with subsamples of varying numbers of cells per tumor, measuring reconstruction error of our SD+CD+GD algorithm with the weighted matching algorithm. The mean reconstruction errors calculated across 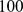 cases for subsamples of 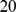, 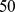, 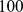, 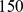 and 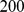 cells were 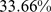 (s.d. 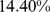), 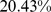 (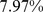), 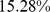 (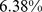), 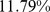 (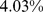), and 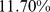 (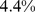) respectively. We can thus conclude that accuracy improves noticeably with increasing numbers of cells to at least 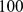 cells per tumor before plateauing at approximately 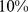 error.

We next assessed numbers of tumors needed to identify meaningful statistically significant properties of tumor classes by analysis of the 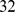 CC paired and primary samples. We randomly subsampled from among the 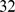 pairs and, for each subsample, calculated the following three tree statistics on progression trees inferred from our SD+CD+GD algorithm:

1. Shannon index based on distribution of cells across different tree levels.
2. Weighted mean depth of the trees.
3. Sum of differences of fractional gain and loss of each gene across the tree edges.

**Figure 9:**
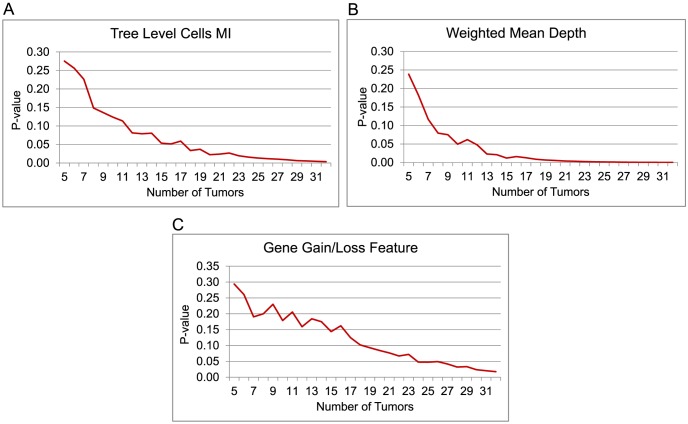
Wilcoxon signed rank test results for separating primary CC samples from the metastases. Wilcoxon signed rank test 1-sided p-values for separating the primary CC samples from the metastases across subsets of increasing numbers of randomly selected tumor samples. For each set of 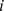 tumors, 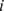 samples were randomly selected from 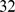 paired CC primary and metastatic tumors with atleast one of each type and then Wilcoxon signed rank test was used to calculate the p-values for separating the primary from metastases based on three different statistics: (A) Shannon index calculated using the distribution of cells across different tree levels, (B) weighted mean depth of the trees and (C) sum of differences of fractional gain and loss of each gene across the tree edges.

We then compared distributions of each statistic on primary vs. metastatic trees by a Wilcoxon signed rank test. As the samples were selected randomly, no ordering among the samples was considered. Figure 9 shows the 1-sided p-values of the three statistical tests when the number of randomly selected samples are increased from 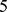 to 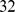. The figure shows that ability to distinguish the two tumor subsets improves with increasing number of tumors. While the threshold for significance varies by statistic, each reaches weak significance (p
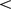
0.05) between 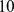 and 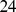 tumors. We can thus conclude that finding reproducible features distinguishing the tree types requires on the order of tens of tumors, at least for the candidate probe sets examined here.

Taken together, these two results demonstrate that building accurate trees on a large enough scale to distinguish meaningfully primary from metastatic trees requires data sets with roughly the order of thousands of single cells (hundreds of cells per tumor for tens of tumors), a scale of data that has so far been achieved only by FISH studies of tumor heterogeneity. We note, however, that one would expect these numbers to vary depending on the degree of tumor heterogeneity, the classes of trees one wishes to distinguish, and the specific markers examined.

## Discussion

This paper has presented novel theory and algorithms for reconstructing evolutionary trajectories of gene copy numbers in solid tumors in terms of a model of tumor evolution incorporating changes at the scale of single gene probes, full chromosomes, or all probes in the genome. We have derived algorithms to reconstruct maximum parsimony sequences of events, and thus estimates of evolutionary distance, between pairs of cells assayed by FISH probes. We have further incorporated these inferences into a method for building phylogenies of hundreds of cells in single tumors. These methods have been added to FISHtrees [46], our software for inferring tumor phylogenies from single-cell copy number data. Experimental results on simulated data confirm the ability of the new methods to improve phylogenetic inference accuracy relative to simpler models by adding CD and GD events that model chromosome-scale and whole-genome copy number changes that are frequently observed in tumor evolution. Application to observed human tumor data shows that these extended evolutionary models are able to yield more parsimonious tree reconstructions and that the resulting trees lead to improved accuracy in prediction tasks related to diagnosis and prognosis.

In future work, we hope to extend the theory developed here to handle even more realistic models and more challenging data types. One important direction will be advancing the theory developed here to improve upon the heuristic approximations used in the Steiner tree inference to better approach the goal of finding globally optimal trees for the most computationally challenging FISH data sets. The evolutionary models, likewise, might be further extended to go beyond the three mutational event types considered here to better approximate the numerous distinct mutational mechanisms by which copy number profiles of tumor cells might evolve. The data sets studied here do not include geographical information about locations of individual cells in the tumor, but other data sets for analyzing tumor heterogeneity do include such geographical information [38], [68]. We expect it would be interesting to construct phylogenies with distance functions that combine spatial distance in three dimensions with combinatorial distance measures between the cell count patterns, as we have studied here. Further, while FISH for the moment retains a unique advantage in the large number of cells it can profile, one can reasonably anticipate that single-cell sequencing will eventually become practical for comparable cross-tumor studies. There would thus be value in extending the theory developed here to single-cell sequencing data, a goal that would pose substantial algorithmic challenges due to the much larger number and variety of markers it can reveal as well as the more complicated error models it would entail. Finally, we hope to make more use of these single-tumor phylogenetic models in clinically relevant prediction tasks and further explore the biological insights one can gain from more accurate tumor phylogenies.

## Methods

Our main theoretical result is a method for inferring minimum distances between two states within a copy number phylogeny when duplication/loss of single genes (SD), duplication/loss of all genes on a common chromosome (CD), and duplication of all genes in the full genome (GD) events are possible. We first establish some mathematical results and then develop an algorithm for accurate distance computation. This algorithm then becomes a subroutine in a heuristic Steiner tree algorithm for inferring copy number phylogenies in the presence of SD, CD, and GD events. We introduce some notation required for specifying and proving the theoretical results:

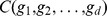
: A set of copy numbers of one or more genes 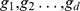, which we call a “configuration”. When 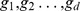 are clear from the context, we use 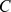 as shorthand.

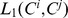
: 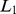 or rectilinear distance between two configurations 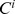 and 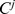.

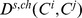
, 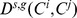, 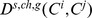: Distance between two configurations 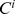 and 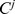 when considering SD+CD (s,ch), SD+GD (s,g), or SD+CD+GD (s,ch,g) events, respectively.

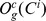
, 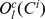, 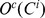: Operations corresponding to single chromosome (CD) events corresponding to either gain (g), loss (l), or either (no subscript) of all genes belonging to the same chromosome 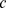 from starting configuration 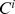, while keeping the copy numbers of genes on other chromosomes unchanged.

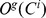
, 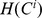: Operations corresponding to doubling (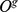) or halving (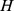) counts of all genes in configuration 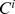. In the case of halving, it is assumed that all genes in 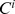 have even counts.
6. *even*, *odd* configuration: A configuration (copy number profile) 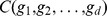 is denoted an *even* configuration if 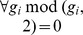. Otherwise, it is denoted an *odd* configuration.

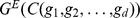
: The set of “nearest even” values for each 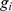 in 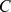, i.e., if 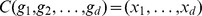 then 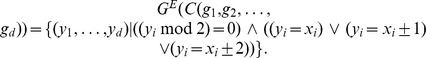 For example, 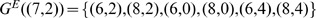.
8. An operation 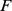 is *valid* on a configuration 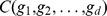 if 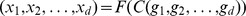 satisfies 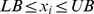 for all 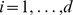 given predefined lower-bound LB and upper-bound UB. Otherwise, 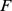 is *invalid* on 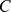. LB = 0 and UB = 9 is used in the sofware, but the theory only requires that UB 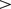 LB.
9. A sequence of operations 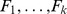 is *boundary-sensitive* on configuration 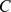 if 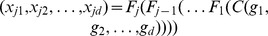 satisfies 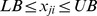 for all 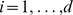 and 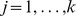. We use *boundary-insensitive* to refer to a sequence on which this condition has not been checked.

### Progression model considering SD and CD events

**Figure 10:**
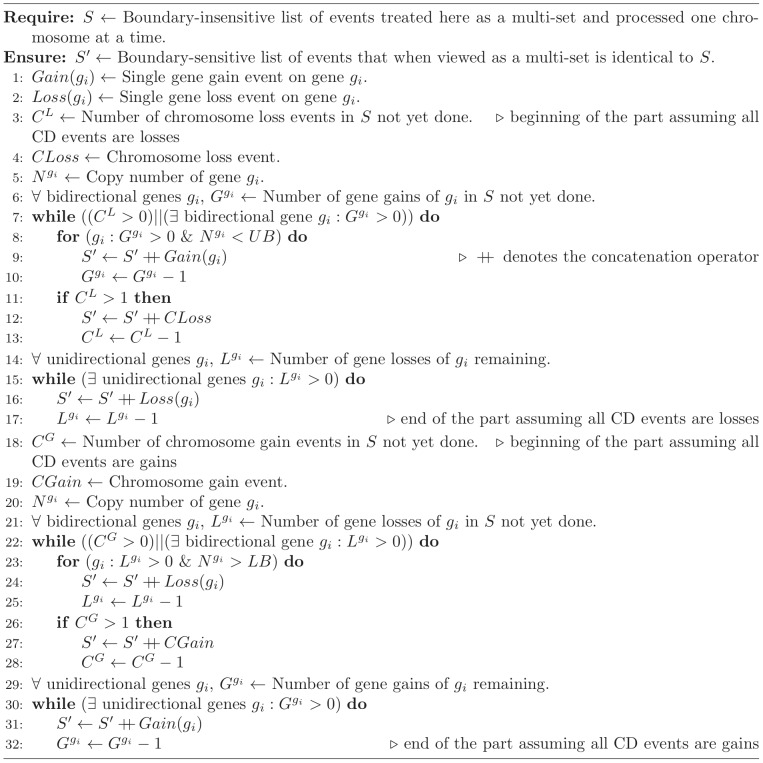
Algorithm 1 pseudocode. Algorithm 1 converts a set of boundary-insensitive events to boundary-sensitive events; lines 3–17 are used for chromosomes on which all CD events are losses and lines 18–32 are used for chromosomes on which all CD events are gains.

We develop the theory for inference of the Steiner (unsampled or extinct cell configurations) nodes in the paths formed by the sequence of gene copy number gains and losses from an initial configuration 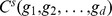 to a final configuration 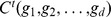. We first extend the prior theory to account for SD and CD events. Our model assumes that on division of a tumor cell, the configuration can change either by gain or loss of one copy of a single gene (SD event) or by gain or loss of one copy of each gene on a single chromosome (CD event). For example, a configuration of four genes 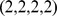 with the first two genes on the same chromosome might evolve in a single mutational event to 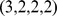 by an SD event or to 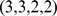 by a CD event. We propose Algorithm 1, provided in Figure 10, to calculate the minimum number of steps required to transform 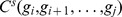 into 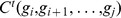 considering SD and CD events, where, without loss of generality, we assume that the genes on a common chromosome have consecutive indices 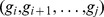 in 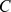. Algorithm 1 also identifies a minimum-length sequence of events, although this sequence is not necessarily unique. For example, if there are four genes on one chromosome and we want to get from configuration 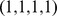 to configuration 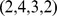, then a shortest sequence of SD and CD events would be CD to 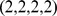, SD to 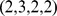, SD to 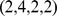, and SD to 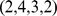. Other orders of the same four events are also possible.

The above example focuses on a single chromosome because as explained below, the problem of finding the shortest SD+CD path can be solved one chromosome at a time. We begin by establishing the following lemmas:

#### Lemma 1

*A minimum-length boundary-insensitive sequence of CD and SD events cannot have both a gain of chromosome* 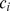 *and a loss of the same chromosome* 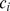 *.*

*Proof.* By contradiction. Suppose 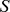 is a sequence of events that has both a gain and a loss of the same chromosome. Then removing one gain and one loss produces a new sequence that is 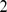 shorter and has the same final state.

#### Lemma 2

*For any gene* 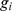 *, a minimum-length boundary-insensitive sequence of events cannot have both a gain of* 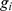 *and a loss of* 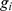 *.*

*Proof.* By contradiction. Suppose 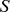 is a sequence of events that has both a gain of 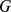 and a loss of 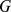. Then removing one gain and one loss produces a new sequence that is 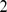 shorter and has the same final state.

#### Lemma 3

*The following sequence of events describes a minimum-length boundary-insensitive sequence of SD and CD events for transforming* 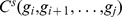 *into* 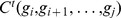 *:*

1. *Perform CD events in arbitrary order starting from* 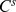 *so that each successive event decreases the* 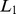 *distance between the intermediate configurations* 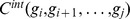 *and* 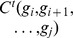 *until any further CD event will increase the* 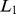 *distance. We define the final configuration reached after this step to be* 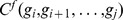 *.*
2. *Perform SD events in abitrary order starting at* 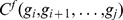 *so that the* 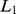 *distance between* 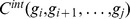 *and* 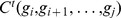 *decreases on each step until the distance becomes zero. The total number of events required will be* 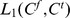 *.*

*Proof.* Since the sequence of events is boundary-insensitive and addition is commutative, we can change the order of events without changing the endpoints or the cost. Therefore, we assume that all CD events precede all SD events. The construction of the above sequence of the events ensures that it uses a maximum number of possible CD events. If we denote the number of genes on the common chromosome by 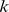 and the number of CD events by 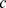, then the total number of events required is 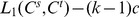. If there exists a shorter sequences of events to transform 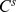 to 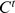, then that sequence must have a larger number 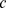 of CD events, which is contradicted by the construction. Thus, the number of events is minimized.

The above lemmas show how to construct a minimum-length boundary-insensitive sequence of events. We now establish that this sequence can be used to derive a minimum-length boundary-sensitive sequence of events:

#### Lemma 4

*For any boundary-insensitive minimum-length sequence of SD and CD events* 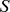 *transforming* 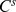 *to* 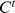 *, there exists a boundary-sensitive sequence of SD and CD events* 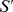 *such that* 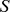 *and* 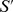 *have equal length.*

*Proof.* We analyze one chromosome at a time because in this section the events on different chromosomes are independent. By Lemma 1, on any specific chromosome all the CD events are gains or all the CD events are losses. We analyze in detail the case in which all CD events are losses; the case of all gains is symmetric.

The proof is constructive. Specifically, we will show that the upper part of Algorithm 1 will transform a boundary-insensitive 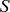 to a boundary-sensitive 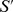 of equal cost solely by reordering events. Without loss of generality, suppose the only CD events in 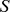 are chromosome losses. There is a symmetric algorithm, shown as the lower part of Algorithm 1, for the case where all the chromosome events are gains. We add the following definition:

A gene 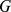 is defined as unidirectional with respect to 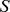 if there are no gains of 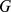 in 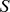. A gene 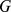 is defined as bidirectional with respect to 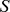 if 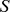 includes gains of 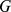. For unidirectional genes, the order of chromosome losses and gene losses can never cause a boundary to be crossed because the copy numbers are monotonically decreasing. The situations we need to avoid are:

1. A bidirectional gene 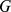 has copy number UB and the next operation affecting 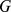 is a gain of 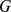.
2. A bidirectional gene 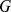 has copy number LB and the next operation affecting 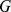 is a chromosome loss.

Chromosome gains are excluded by Lemma 1 and our assumption without loss of generality that all CD events are losses. Gene losses for bidirectional genes are exluded by Lemma 2.

To prove correctness of the algorithm, we note that 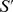 can never cross LB for the unidirectional genes because their net loss equals their total loss. 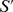 can never cross LB for the bidirectional genes, because when their copy number is at LB, a gene gain must still be pending and the gene gains alternate in the first while loop until no chromosome losses or gene gains are remaining. 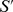 can never cross UB for the unidirectional genes because they have only losses. 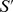 can never cross UB for the bidirectional genes because of the test 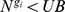 (line 8) before any gene gain is done. Further, all the chromosome losses will be used because one chromosome loss happens on each pass through the first while loop, if any chromosome losses remain. All gene gains in 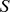 will be used in the first while loop because the net change for any gene must keep its copy number below UB. All the gene losses for the unidirectional genes are used in the second while loop. The unordered set of events and total change in each gene is thus preserved between 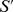 and 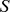, while 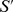 guarantees that the sequence is boundary-sensitive.

We use the preceding result to derive the main theorem of this section, which estabishes a method to find a minimum-length sequence of SD and CD events transforming 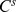 to 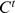. As in the proof of Lemma 4, we can consider each chromosome separately since each SD and CD event affects only one chromosome.

#### Theorem 5

*Assume we partition the gene list by chromosomes such that each chromosome* 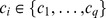 *corresponds to a consecutive subset of genes* 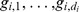 *. Further define* 
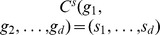
 *and* 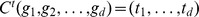 *. Then we can construct a minimum-length boundary-sensitive sequence of events transforming* 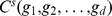 *to* 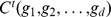 *by constructing a minimum-length boundary-sensitive sequence of events* 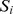 *transforming* 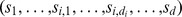 *to* 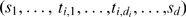 *for each chromosome* 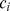 *and interleaving each* 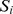 *in arbitrary order.*

*Proof.* The distance function can be decomposed into individual parts for genes belonging to distinct chromosomes as follows:
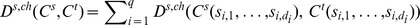



Because the distance cost can be decomposed in this way and each CD or SD event contributes to only a single term of the outer sum, we can minimize the cost of events for each chromosome independently and combine the events from distinct chromosomes in arbitrary order without changing the value of the objective function. Likewise, since these each chromosome affects a disjoint subset of genes, boundary-sensitive sequences for each chromosome will yield a boundary-sensitive sequence across all genes.

### Progression model combining SD, CD and GD events

We now extend the theory from the prior section to include SD, CD, and GD events. We assume in the proofs and discussion below that 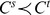, where 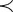 denotes lexicographical ordering. This assumption reduces the number of cases in several proofs. If instead, 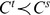, the proofs are identical or symmetric except that GD events may be used in the wrong direction (halving instead of doubling). The use of halving events is corrected heuristically by a procedure of subtree pruning and regrafting at line 24 of the pseudocode of Algorithm 3, described below, and in FISHtrees. We will produce the complete proof by deriving a series of lemmas for three cases that together will cover all possible 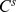 and 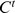:

#### Lemma 6

*For an an even configuration* 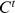 *, if there exists an optimal sequence of copy number change events from* 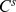 *to* 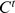 *composed of one or more SD and CD events and a single GD event, then the following sequence of events is of minimum length:*

1. *SD and CD events to transform* 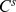 *into* 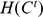 *, constructed as described in the first named subsection of Methods*
2. *A single GD event to transform* 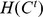 *into* 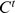 *.*

*Proof.* We prove the statement by considering the three different ways that can be used to transform 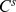 to 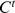 using single GD and multiple SD and CD events. The statement of the lemma presents one case and the remaining two possibilities are as follows:

1. A single GD event to transform 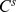 into 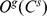 and then multiple SD and CD events to transform 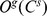 into 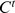.
2. Multiple SD and CD events to transform 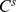 to an intermediate configuration 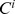, a single GD event to transform 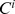 into 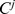, and multiple SD and CD events to transform 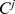 into 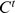.

We show that for either of these alternative cases, we can produce a sequence satisfying the conditions of the lemma with equal or smaller length. For the first case, we have to show that
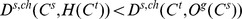



It can be seen that 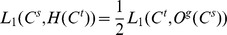



If all genes are located on distinct chromosomes, then, 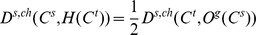
and the claim follows directly.

Now, assume the genes are partitioned into sets of chromosomes such that each chromosome 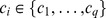 corresponds to a consecutive subset of genes 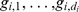. We focus on a specific chromosome 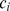 and consider the problem of updating just genes of that chromosome from their values in 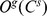 to their values in 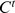. Either zero or a positive even number of CD events must be performed to convert these genes from 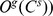 to 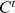 and along with zero or a positive even number of SD operations on each gene. If an odd number of CD operations are performed on 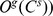, then we get an odd configuration and at least one or an odd number of SD operations must be performed on each gene of this odd configuration to convert it to the even configuration 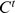. But a combination of single SD operations acting on each of the individual genes in 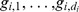 has the same effect as a single CD operation on chromosome 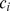 and this combination therefore cannot be minimal. Therefore, the number of CD operations is even. If a total of 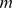 CD operations and 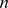 SD operations are needed to convert 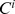 to 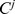, then a total of 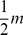 CD operations and 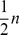 SD operations are needed to convert 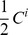 to 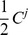. So, 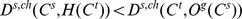



For alternative 2, we can write the distance function as: 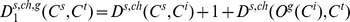



The distance function for our proposed optimal sequence can be written as: 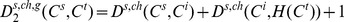



As shown for alternative 1, we can write: 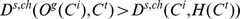
which implies 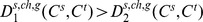.

#### Lemma 7

*For an odd configuration* 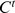 *, if the optimal sequence of copy number change events from* 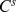 *to* 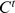 *is composed of one or more SD and CD events, followed by a single GD event, followed by one or more SD and CD events, then the configuration from which the final set of SD and CD events take place is a member of* 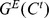 *.*

*Proof.* We denote the intermediate configuration following the GD event to be 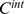. We will show by contradiction that if there exists any optimal sequence of events for which 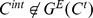 then there must exist an alternative, shorter sequence of events. Define the full sequence of events from 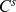 to 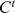 to be 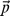, subdivided into the subsequences 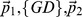. First, we note that if there is any duplicated event in 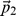 then we can construct a more parsimonious solution by replacing the duplicate in 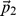 with a single copy of the event in 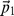. Therefore, no event appears more than once in 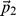. There are exactly two SD and CD events that can increase the count of any given probe (SD of that probe or CD of its chromosome) and similarly exactly two events that can decrease the count of any probe. Thus, no probe's value changes by more than 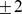 in the transition from 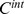 to 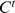 in 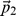. Finally, we note that since 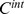 immediately follows a GD event, it must be an even configuration. Together, these assertions establish that 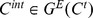 for any optimal path 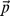.

#### Lemma 8

*For an odd configuration* 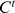 *, if the optimal sequence of copy number change events from* 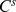 *to* 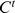 *is composed of one or more SD and CD events and a single GD event, then the optimum sequence of events follows the following path:*

1. *Generate* 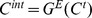 *.*
2. *SD and CD events to transform* 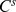 *into* 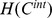 *.*
3. *A single GD event to transform* 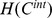 *into* 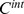 *.*
4. *SD and CD events to transform* 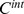 *into* 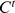 *.*

*The optimal sequence is an element of the set of sequences generated using this procedure.*

*Proof.* The proof follows from application of Lemma 6 and Lemma 7. As 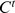 is an odd configuration, the final step cannot be a GD event. So, the last steps have to be a combination of SD and/or CD events; in that case, Lemma 7 shows that the configuration reached as a result of GD must be a member of 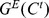, which we denote by 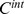. Lemma 6 shows that to reach any member of 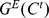, which are even configurations, the optimal sequence of events is to generate SD and CD events to transform 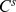 into 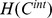 first and then to perform a GD event to transform 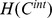 into 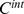. This sequence of events matches the sequence proposed in the lemma.

**Figure 11:**
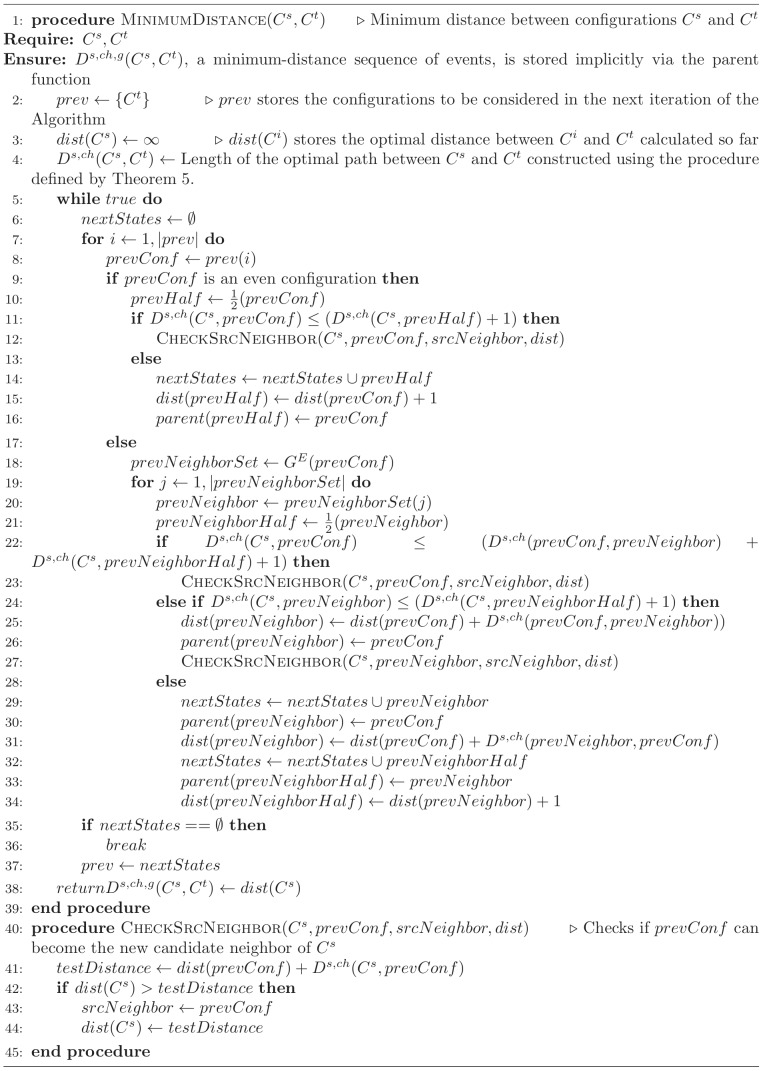
Algorithm 2 pseudocode. Algorithm 2 finds the shortest directed distance between two configurations using SD, CD, and GD events.

The above lemmas allow us to derive Algorithm 2 to transform 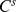 to 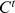 using a minimum-length combination of SD, CD and GD events. The pseudocode of Algorithm 2 is presented in Figure 11. To illustrate the algorithm, suppose 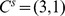 and 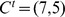, where we will assume we have two probes on a single chromosome. Since 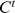 is an odd configuration, we first generate its nearest even neighbors 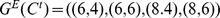 and calculate 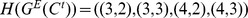. The algorithm tests for two stopping conditions by which a solution can be constructed (lines 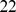 and 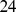 in Algorithm 2), neither of which applies to any of the solutions at this point. 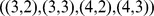 are therefore considered for the next iteration. 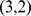, 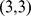, and 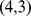 are odd configurations, so we generate their neighbor sets 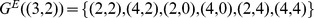, 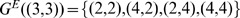, and 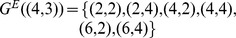. One stopping condition is satisfied for each of the elements of these neighbor sets, so 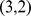,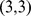, and 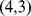 are each considered in turn as the next candidate neighbor. 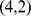 is an even configuration, so we only need to consider one possible stopping condition (line 11), which it satisfies, so it is also considered as a possible next candidate neighbor. Among the four possibilities, we will conclude that using 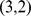 as the immediate neighbor will lead to the smallest possible number of steps when accumulating SD+CD events from 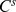 to the candidate, a single GD event from the candidate to its double, and SD+CD events from that double to 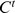. Following some postprocessing updates (procedure CheckSrcNeighbor), the algorithm computes a minimum-length solution of 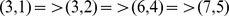 and returns the corresponding length 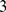.

Algorithm 2 satisfies the following theorem, which constitutes the major result of this section:

#### Theorem 9

*Algorithm 2 returns the minimum distance between two configurations* 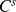 *and* 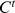 *, where* 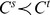 *.*

*Proof.* We use induction on the minimum number of steps to get from 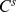 to 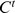, which we denote by 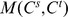.

**Base case.** For the base case, we have 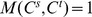. We must consider two sub-cases: (i) 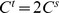 and (ii) 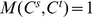. For case (i), 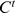 is an even configuration. The condition at line 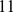 in Algorithm 2 fails and 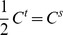 is considered for the next iteration. In the next iteration, if 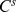 is an even configuration then the condition at line 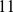 is now satisfied and 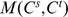 is assigned the value 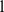 in CheckSrcNeighbor procedure called at line 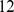 in the main procedure. If 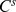 is an odd configuration, then the condition at line 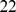 is satisfied for each of the even neighbors of 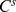 and 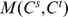 is assigned the value 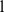 in the CheckSrcNeighbor procedure called at line 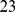. For case (ii), one of the conditions at line 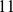 or line 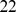 is satisified in the first iteration of the algorithm depending on whether 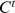 is an even or odd configuration and 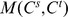 is assigned the value 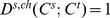 at line 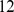 or 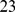.

**Induction step.** For the induction hypothesis, we assume that the the algorithm uses the minimum number of steps for all cases where 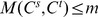. Then, suppose that an adversary selects an example that has complexity 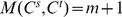. Let us assume that the penultimate configuration in the optimal solution is 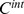. If 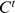 is an even configuration, then it can be reached from 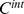 by using (i) a GD event, (ii) an SD event, or (iii) a CD event. According to the induction hypothesis, for each of these cases, Algorithm 2 uses the minimum number of 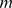 steps to generate 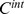 from 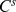. If there is at least one GD event in the optimal solution, then Algorithm 2 first calculates 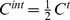. The induction hypothesis ensures that 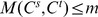 and thus, Algorithm 2 returns a solution with a maximum length of 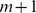. If there is no GD event in the optimal solution from 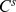 to 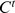, then Algorithm 2 uses the procedure described in the first named subsection of Methods to calculate the optimal path from 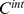 to 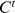 and combining it with the optimal solution from 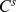 to 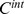, it returns the optimal path between 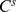 and 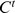. Now, if 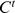 is an odd configuration, then going from the penultimate configuration 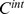 to 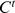 can only be achieved using either an SD or a CD event. For odd 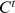, Algorithm 2 first generates its even neighbors 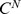 which are steps 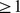 from 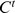. If 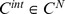, the proof follows directly from the inductive hypothesis. If 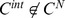, then there is a 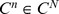 such that 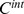 is located on the optimal path between 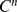 and 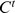 formed using SD and CD events only. If 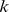 is the total number of genes with odd copy number values in 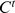, then 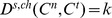 and 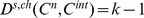. Using the induction hypothesis, we can write,
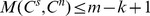



As Algorithm 2 uses the procedure described in the first named subsection of Methods to construct the optimal path between 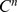 and 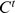, we can see that it returns a path with 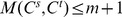.

### Runtime analysis of Algorithm 2

We provide an upper bound on the runtime of Algorithm 2 as a function of the number of genes 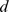 and their copy numbers. Considering all three events, where 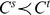, the maximum number of doublings required is 
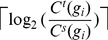
, where 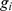 denotes the copy number of the first gene where 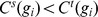 and 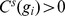. At each stage of the algorithm, the maximum number of nodes generated as a result of a 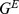 operation is 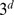. 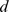 SD and CD events are used to create each of those 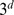 nodes in the case of an odd configuration. So, the maximum number of required 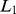 operations is 
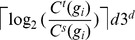
. Therefore, the number of operations performed during the execution of Algorithm 2 is 
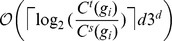
.

### Generating tumor phylogenies

**Figure 12:**
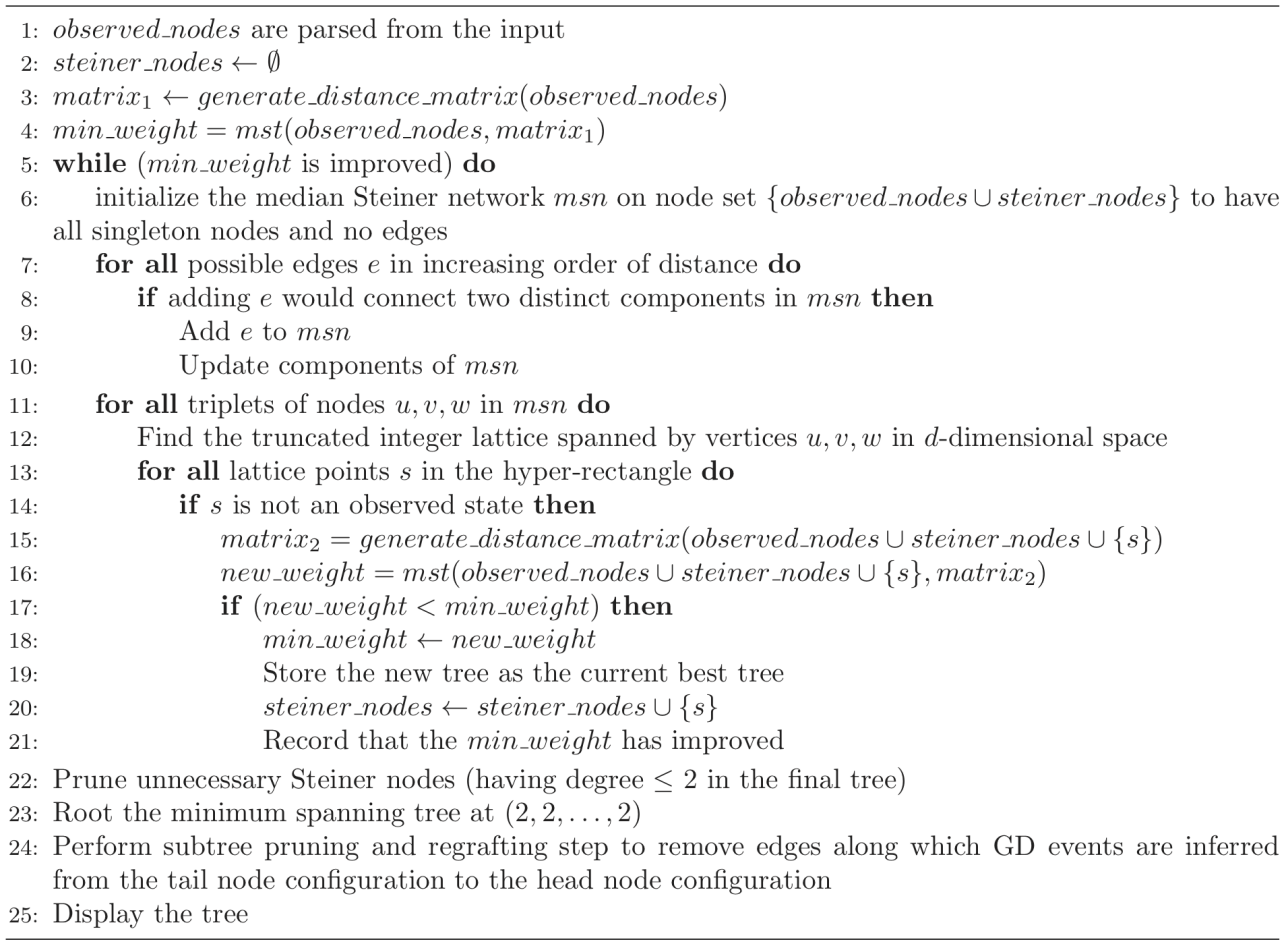
Algorithm 3 pseudocode. This figure provided the main steps in the algorithm to generate tumor progression trees; generate_distance_matrix uses Algorithm 2 on each distinct pair of nodes in the set of nodes it is passed. To compute Minimum Spanning Tree (function *mst* called at lines 4 and 16), we implemented Prim's algorithm.

We implemented Algorithm 2 and integrated it with our approximate median-joining-based algorithm from our prior SD-only FISHtrees [46] code. The key steps of this algorithm are summarized in Algorithm 3 (Figure 12), which we describe at a high level here. The phylogeny algorithm first relies on Algorithm 2 to derive a matrix of pairwise distances between observed cell configurations, which are treated as states on a truncated integer lattice of dimension 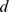 with a maximum value (UB) set to 9 in the current code. It then repeatedly samples triplets of nodes, identifying as potential Steiner nodes those that agree in each dimension with at least one of the triplet. Those Steiner nodes that lead to reduced minimum spanning tree cost are added to the node set, with the process is repeated until there is no further improvement. Finally a series of post-processing steps are performed to prune Steiner nodes that are not needed for the final tree and to apply subtree regrafting to correct for a potential source of suboptimality arising from the fact that the core phylogeny algorithm assumes symmetric distances but GD operations are asymmetric.

### Inferring tumor phylogenies using Neighbor Joining (NJ) and Maximum Parsimony (MP) methods

Neighbor Joining (NJ) and Maximum Parsimony (MP) methods have been commonly used for building single-tumor phylogenies [16], [54] and we therefore compared their accuracy to that of our own methods in inferring copy number phylogenies. We applied these two traditional phylogenetic tree building methods to build tumor progression trees using the individual copy number profiles as taxa and compared them with the trees built using our algorithms. We used implementations of both approaches in MEGA version 6 [69]. For NJ, we used Euclidean distances between cell copy number profiles to build the pairwise distance matrix. For MP, we treated copy number profiles of the genes in individual cells as sequences of arbitrary phylogenetic characters. We used the “Close-Neighbor-Interchange on Random Trees” search method. For the parameters “Number of Initial Trees” and “MP search level”, we used values of 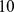 and 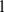 respectively.
